# Czech nationwide screening for Fabry disease in patients on maintenance dialysis: a call for evaluation of population-enriched *GLA* gene variants of uncertain significance

**DOI:** 10.1093/ckj/sfaf167

**Published:** 2025-05-28

**Authors:** Ivan Rychlík, Lidmila Francová, Gabriela Dostálová, Marie Pešková, Satu Pešičková, Miroslav Ryba, František Švára, Zuzana Nemcová, Sylvie Dusilová Sulková, Ondřej Viklický, Jana Reiterová, Michaela Ságová, Jan Geryk, Karolína Krátká, Vladimír Tesař, Aleš Linhart, Milan Macek

**Affiliations:** Department of Internal Medicine, Third Faculty of Medicine, Charles University, Prague and University Hospital Královské Vinohrady, Prague, Czech Republic; Department of Internal Medicine, Third Faculty of Medicine, Charles University, Prague and University Hospital Královské Vinohrady, Prague, Czech Republic; Second Department of Medicine, Cardiology and Angiology, First Faculty of Medicine, Charles University, Prague, and General University Hospital, Prague, Czech Republic; Department of Internal Medicine, Hospital, České Budějovice, Czech Republic; Dialysis Unit, B. Braun Avitum Ohradní, Prague, Czech Republic; Dialysis Unit, Liberec Regional Hospital Liberec, Czech Republic; Dialysis Unit, B. Braun Avitum Teplice, Teplice, Czech Republic; Dialysis Unit, Hedica, Boskovice, Czech Republic; Department of Nephrology, Faculty of Medicine, Charles University and University Hospital, Hradec Králové, Czech Republic; Department of Nephrology, Institute of Clinical and Experimental Medicine, Prague, Czech Republic; Department of Nephrology, First Faculty of Medicine, Charles University, Prague, and Faculty General Hospital, Prague, Czechia; Fresenius Medical Care – DS, Prague, Czech Republic; Department of Biology and Medical Genetics – Second School of Medicine Charles University and Motol University Hospital, Prague, Czech Republic; Department of Internal Medicine, Third Faculty of Medicine, Charles University, Prague and University Hospital Královské Vinohrady, Prague, Czech Republic; Department of Nephrology, First Faculty of Medicine, Charles University, Prague, and Faculty General Hospital, Prague, Czechia; Second Department of Medicine, Cardiology and Angiology, First Faculty of Medicine, Charles University, Prague, and General University Hospital, Prague, Czech Republic; Department of Biology and Medical Genetics – Second School of Medicine Charles University and Motol University Hospital, Prague, Czech Republic

**Keywords:** dialysis, end-stage kidney disease, Fabry disease, *GLA* gene, screening

## Abstract

**Background:**

Fabry disease (FD) is a rare disorder caused by variants in the GLA gene encoding α-galactosidase A (GALA), leading to end-stage kidney disease (ESKD), among other health issues. The 2002 Czech nationwide FD screening in ESKD found undiagnosed cases in dialysis patients by examining GALA activity in dried blood spots (DBS).

**Methods:**

The second nationwide FD screening (2016–2018; 21-month study) in ESKD patients on maintenance dialysis therapy (MDT) included 112 Czech dialysis units to assess country-wide FD diagnostic guidelines' efficacy in reducing its underdiagnosis. This involved GALA activity and/or lyso-Gb3 levels with *GLA* sequencing in positive males, the latter applied first in all females, followed by lyso-Gb3 examination in variant-positive cases. Screening-positive cases were referred to the FD center for follow-up and *in vitro* studies.

**Results:**

The 6352 screened cases represent 93.9% of all MDT patients within the study duration. Eight *GLA* variants were identified in 39 patients, of which seven were in 35 ESKD cases classified as likely benign (LB), with normal lyso-Gb3 levels in all subjects. Four patients (three males with reduced GALA activity and one sequencing-positive female) bear a “hot variant of uncertain significance” (VUS) c.1181T>C(p.Leu394Pro), significantly enriched compared to the general population, suggesting its association with FD.

**Conclusions:**

This is one of the largest FD screening schemes in a European ESKD cohort. Subsequent *in vitro* studies proved that the hot VUS is linked to alternative FD pathogenesis, thereby substantiating the utility of combining biomarkers and sequencing/bioinformatics in FD screening. The broad application of FD diagnostic guidelines has reduced its underdiagnosis in ESKD.

## Graphical Abstract

**Graphical Abstract:**
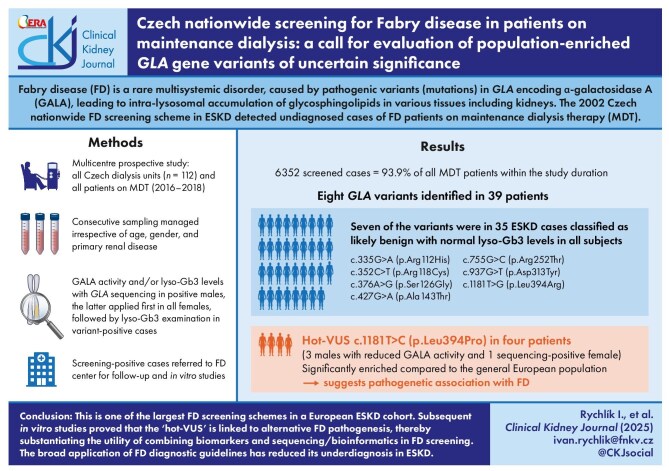

KEY LEARNING POINTS **What was known:**

- Fabry disease (FD) is a rare multisystemic disorder caused by pathogenic variants (mutations) in the GLA gene encoding α-galactosidase A (GALA). These mutations lead to intralysosomal accumulation of glycosphingolipids in the kidneys, resulting in end-stage kidney disease (ESKD). However, GLA involvement in the pathogenesis of ESKD is not fully elucidated.
- The 2002 Czech nationwide FD screening scheme in ESKD detected undiagnosed cases of FD in patients on maintenance dialysis therapy (MDT) by examining only GALA activities in dried blood spots.
- Hence, to assess the efficacy of country-wide guidelines in the last decade and to establish the current prevalence of undiagnosed FD among MDT patients, we carried out a second nationwide screening (*n* = 6352 ESKD cases; years 2016–2018) using a combination of two biomarkers (decreased GALA activity and/or increased lyso-Gb3 levels) in males followed by *GLA* sequencing in screening positives, with the latter applied first in females followed by lyso-Gb3 levels in variant-positive cases.

**This study adds:**

- Eight GLA variants were identified in 39 patients, of which seven were in 35 ESKD cases and classified as likely benign (LB), with normal lyso-Gb3 levels in all subjects. Four patients (three males with reduced GALA activity and one sequencing-positive female) bear a ‘hot VUS’ c.1181T>C (p.Leu394Pro), significantly enriched compared to the general population, suggesting its association with FD.
- Subsequent complementary *in vitro*, clinical, and laboratory studies corroborated the suggestive pathogenic association of the p.Leu394Pro with FD through alternative pathogenesis via altered proteostasis of GALA in chronic endoplasmic reticulum stress and unfolded protein response in expressing cells.

**Potential impact:**

- To date, this is one of the largest population-specific FD screening schemes for ESKD in a representative, homogeneous European population.
- This study demonstrates the utility of combining biomarkers and sequencing/bioinformatics in FD screening in ESKD.
- Since 2006, we have increased awareness of complex FD symptomatology and fostered long-term nationwide multidisciplinary collaboration, representing an optimal strategy for diminishing FD underdiagnosis in ESKD.

## INTRODUCTION

Fabry disease (FD; MIM 301 500; ORPHA 324) is a rare monogenic disorder caused by pathogenic variants in the *GLA* gene (MIM 300 644; henceforward *GLA*) that encodes the α-galactosidase A enzyme (GALA). Deficient GALA activity leads to intralysosomal accumulation of glycosphingolipids, predominantly globotriaosylceramide (lyso-Gb_3_), in various tissues, causing gradual structural and functional cellular damage [1, 2]. FD is an X-linked disorder, i.e. hemizygous males are usually more severely affected than heterozygous females, where FD's severity largely depends on the X chromosome inactivation [3].

The classical form of FD is characterized by multiple organ involvement and age-dependent manifestations [4]. Renal involvement is commonly observed and results in progressive chronic kidney disease (CKD), eventually requiring kidney replacement therapy (KRT) [5]. Approximately 14% of male and 2% of female patients suffering from FD require KRT [6].

Multiple screening studies in patients with end-stage kidney disease (ESKD) have shown variable prevalence of previously unrecognized FD cases with a mean prevalence of *GLA* variants of 0.42% in males and 0.68% in females [7], similar to other reports [8, 9] (see the Supplementary Material). Notably, despite the minimal diagnostic yield, cascade family screening in discovered cases often allows the identification of additional FD patients and their at-risk genetic relatives, as in our 2002 Czech study, which utilized the examination of GALA in dried blood spots (DBS) [10].

Recently, GALA activity assays using DBS have become widely available for reliable screening of FD in males. Moreover, since 2010, the lyso-Gb3 biomarker has been introduced into FD diagnostics [11] alongside GALA activity measurements, increasing the screening specificity and sensitivity in classical/late-onset forms of FD. However, in females, the GALA activity is often inconclusive. Therefore, FD screening requires sequencing the entire *GLA* coding sequence using DBS samples followed by examining lyso-Gb3 levels whose elevation is associated with pathogenic *GLA* variants [12].

In the last decade and a half since the first nationwide screening, we have developed FD screening recommendations that have been uniformly applied in Czechia at all dialysis units.

Therefore, the study hypothesis of the second Czech nationwide screening of FD in ESKD patients on MDT was not only to (i) assess the efficacy of applied FD screening recommendations, but also to analyze (ii) whether the combination of two FD-associated biomarkers (i.e. GALA and lyso-Gb3 level examination) together with *GLA* sequencing/advanced bioinformatic analyses could identify additional/atypical cases of FD that could have been missed by the previous FD screening methodology and assure an equitable screening approach in male and female subjects with ESKD.

## MATERIALS AND METHODS

### Study design

This multicentre observational study included all dialysis units (*N* = 112) (www.nefrol.cz/mapa). Hemodialysis (HD) and peritoneal dialysis (PD) patients were included in the sampling period, ranging from 1 September 2016, to 30 May 2018 (21 months). The sampling procedure was performed consecutively, comprising all regions of the country and enrolling subjects currently in the MDT. Adult patients [4] were enrolled irrespective of age, sex, and primary renal disease (PRD). No further exclusion or inclusion criteria were applied.

The resulting screening cohort was prospective, longitudinal, and representative character. Altogether, 6352 cases were screened (henceforward referred to as the “study cohort”; SC) (Table 1) and represented 93.9% of prevalent patients on MDT as of 31 December 2017 (*N* = 6768 patients, i.e. prevalence of 639 per million of the general population) [13]. The SC had a male-to-female ratio (MFR) of 37%/63%, with a mean age of 68.5 ± 14.2 years.

**Table 1:** Overview of the study cohort.

Year	2016	2017	2018
No. of screened patients (total 6 352)	2 585	3 091	676
No. of prevalent HD patients	6 310	6 373	6 631
No. of prevalent PD patients	429	395	359

Maintenance dialysis patients screened within the 21-month study period (ranging from 1 September 2016 to 30 May 2018; a total of 21 months), including numbers of HD and PD prevalent patients on 31 December coinciding with the study period (2016–2018) drawn from the RDP database.

All patients in the SC have been treated according to the national standard of dialysis care [14, 15] and provided written informed consent before enrollment on the condition of their pseudonymization. This study was conducted under the auspices of the Czech Society of Nephrology (CSN; www.nefrol.cz).

### Data collection

All laboratory and clinical data gathered from the SC were collected from the Registry of Dialysis Patients (RDP) database [13] managed by the CSN, curating all Czech patients within the last few decades. The data included demographic characteristics (age and sex), PRD listed as the cause of ESKD, and additional data concerning the presence of comorbidities [13]. The RDP database also includes information on MDT, of which most are on HD, and a subset of ∼6% are on PD. Hence, in RDP, we considered incident cases as those who started regular dialysis during the entire calendar year, while prevalent cases were those registered as of the year's last day. Due to the involvement of all dialysis centers, we could not achieve an optimal (i.e. 100%) sampling of the prevalent ESKD population since some of them were, e.g. hospitalized or temporarily attended other dialysis centers within the study period. In addition, ∼23% of all ESKD cases died, and ∼8% were transplanted in a given year [13]. Therefore, based on the week-to-week variability of the continuously dialyzed/sampled cases, together with the inability to test all cases at each of the participating units, for statistical analyses, we considered the SC representative since it comprised 93.9% of all prevalent cases within the 2016–2018 period (Table 1). Notably, the *GLA* variant-positive subsets were subtracted to ensure valid and statistical analyses, or MFR was not assessed in a small number of variant-positive cases (see further Results).

### Clinical classification of cases

**Figure 1:**
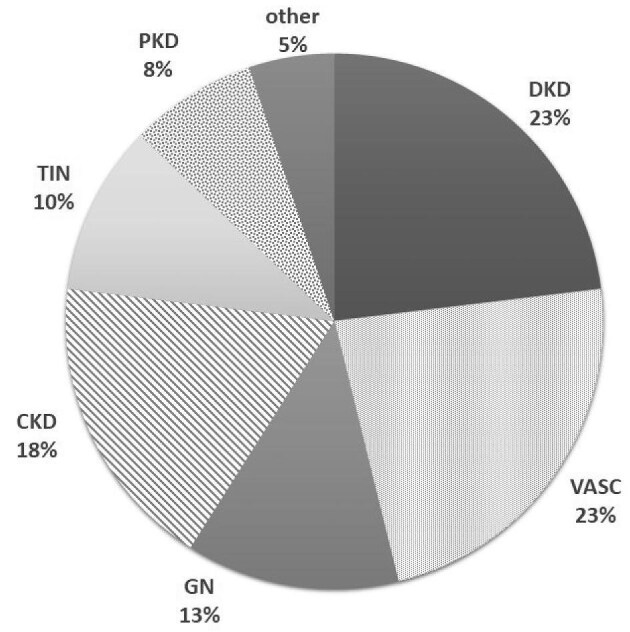
Diagnoses of PRD in the cohort of 39 *GLA* variant-positive cases. PRD diagnoses (using the ICD-10 coding system): diabetic nephropathy (DKD) (ICD: E10-E14, N083; eight cases; ∼23%), renal vascular disease (VASC) (ICD: I12, I70, N28; eight; 23%), chronic kidney disease/chronic renal failure of unspecified origin (CKD) (ICD: N18; i.e. shrunken kidney or CKD of unknown origin; seven; 18%); chronic glomerulonephritis (GN) (ICD: N00-N08; five; 13%), chronic tubulointerstitial nephritis (TIN) (ICD: N11, N16; four; 10%), polycystic kidney disease (PKD) (ICD: Q61; three; 8%), and ‘other’ PRDs (ICD: other NXX; two; 5%).

PRD was coded using the ICD-10 classification, and the attending nephrologist established the diagnosis in individual dialysis units [16]. However, only a minority of PRD cases were biopsy-proven, except cases of glomerulonephritis based on renal biopsy (RB) (Fig. 1).

Follow-up data for all detected *GLA* variants (*n* = 39) were extracted from the RDP database [13], including prospective follow-up of cases and quarterly reports of selected clinical/laboratory data. However, only survival, death, or kidney transplantation (KTx) were used for this analysis with minimum follow-up up to 30 May 2021 (i.e. min., 3 years) in the case of the 2018 datasets and for 5 years for cases screened in 2016. As Czech legislation requires, the follow-up data of the remaining 6313 *GLA* variant-negative cases in the SC could not be utilized because of compulsory case anonymization after being enlisted in the RDP. However, we consider them equivalent to the respective RDP datasets from within the 2016–2018 period due to the high degree of representativeness of the entire SC cohort (see above).

### FD screening

All samples were analyzed using CENTOGENE AG (Rostock, Germany; www.centogene.com). The FD screening algorithm varied by gender: in males, enzymatic GALA activity (via fluorimetry) and lyso-Gb3 concentration (via liquid chromatography-mass spectrometry) were assessed [17]. GALA activity <15.3 µmol/l/h and/or lyso-Gb3 >1.8 ng/ml triggered full *GLA* (NM_000169.2) sequencing. In females, *GLA* sequencing preceded lyso-Gb3 analysis in variant-positive cases. The screening-positive cases were referred to a specialized Prague FD center for complementary *in vitro*, clinical, and laboratory analyses. Details of the statistical and bioinformatics methods are provided in the Supplementary Material.

## RESULTS

### Examination of GALA activity and lyso-Gb3 levels

The mean values of biochemical examinations in all *GLA* variant-positive cases were for the lyso-Gb_3_ (*N* = 39) biomarker 1.13 ng/ml (range 0.8–1.5) and for GALA activity (in 12 males only) was 8.73 µmol/l/h (range 0.6–15.9), which represents the mean enzymatic GALA activity (given as measured/reference value) of ∼57% in the entire male population of the SC (Table 2). Notably, the levels of lyso-Gb3 were normal in all male/female screening-positive subjects. However, three male cases had a mean GALA activity of 25%, while the two additional males with “altered” GALA activity at 68% and 69% were thus indicated for *GLA* sequencing (Table 3).

**Table 2:** Comparison of two *GLA* variant-positive datasets during the study period.

Parameter	Hot VUS *GLA* variant c.1181T>G (p.Leu394Pro) (*n* = 4)	LB *GLA* variants (*n* = 35)	Study cohort (*n* = 6 352)	Incident patient RDP^b^ (*n* = 1 624)	Prevalent patient RDP^b^ (*n* = 6 768)
Male, no. (%)	3 (75.0%)	9 (25.7%)	3 773 (59.4%)	1 013 (62.4%)	4 128 (61.0%)
Female, no. (%)	1 (25.0%)	26 (74.3%)	2 579 (40.6%)	611 (37.6%)	2 640 (39.0%)
Mean age (years)	^a^70.1	^a^71.0	64.6	67.0	65.1
Average dialysis vintage (days)	^a^2133	^a^2947	NAV	NA	1 644
Deceased, no. (%)	^a^1 (25.0%)	^a^14 (40.0%)	NAV	NA	1 538 (22.7%)
KTx, no. (%)	0	^a^3 (8.6%)	NAV	NA	469 (6.9%)
DM, no. (%)	2 (50.0%)	11 (31.4%)	2 877 (45.3%)	663 (39.0%)	2 985 (44.1%)
lyso-Gb_3_ (ng/ml)					
Males	1.23	1.11	NA	NA	NA
Females	1.00	1.12	NA	NA	NA

DM, diabetes mellitus; NA, not applicable; NAV, not available (follow-up data are not available since *GLA* variant-negative cases that had been continuously entered into the RDP had to undergo compulsory anonymization due to the national data-security legislation: see paper); yrs, years;

a Data provided for the follow-up of up to 30 May 2021, (i.e. a minimum of 3 years);

b Data provided for the year 2017 in RDP patients;

There was no difference between the p.Leu394Pro variant subgroup (*N* = 4) and the subgroup of 35 cases with LB GLA variants (*P* < .20377);

**Table 3:** Detected *GLA* gene variants and their bioinformatic variant effect predictions (stratified by patient gender).

*GLA* gene variant	Genome position (chrX)	Ref.	Alt.	gnomAD v.4.1.0 freq.	ACGT.cz	Alpha missense	AMP/ACMG class	Odds ratio	Odds ratio, confidence interval	No.	M/F	Mean age (years)	Mean GALA conc.	Mean GALa activity (%)	Mean lyso-Gb3 (ng/ml)
c.335G>A (p.Arg112His)	101 403 845	C	T	0.0 000 160	0	0.2727	LB	7.22	[0.65, 79.63]	1	F	87.7	NA	NA	1.50
c.352C>T (p.Arg118Cys)	101 403 828	G	A	0.0 006 314	0	0.1053	LB	0.70	[0.17, 2.91]	2	F	76.5	NA	NA	1.15
c.376A>G (p.Ser126Gly)	101 401 803	T	C	0.0 005 453	0	0.0737	LB	0.24	[0.03, 1.74]	1	F	70.5	NA	NA	1.30
c.427G>A (p.Ala143Thr)	101 401 752	C	T	0.0 006 179	0	0.1608	LB	0.14	[0.02, 1.04]	1	F	43.9	NA	NA	1.00
c.755G>C (p.Arg252Thr)	101 398 831	C	G	0.0 000 110	0.000 492	0.058	LB	7.22	[0.65, 79.66]	1	F	79.4	NA	NA	1.30
c.937G>T (p.Asp313Tyr)	101 398 432	C	A	0.0 038 700	0.003 048	0.1505	LB	0.73	[0.5, 1.07]	8	M	71.6	10.30	68	1.13
	101 398 432	C	A	0.0 037 350	0.003 048	0.1505	LB	*	*	20	F	70.8	NA	NA	1.08
**c.1181T>G (p.Leu394Pro)**	**101 397 918**	**A**	**G**	**0.00 000 252**	**0**	**0.3679**	**hVUS**	**17.78**	**[3.26, 97.1]**	**3**	**M**	**70.3**	**3.73**	**25**	**1.23**
	**101 397 918**	**A**	**G**	**0.00 000 123**	**0**	**0.3679**	**hVUS**	*	*	**1**	**F**	**71.0**	**NA**	**NA**	**1.00**
c.1181T>G (p.Leu394Arg)	101 397 918	A	C	0.0 000 000	0	0.1617	LB	4.44	[0.4, 49.01]	1	M	80.5	10.5	69	1.10
**Total**										**39**	**12/27**	**70.9**			**1.13**

ACGT.cz, Czech population genomics database variant frequency irrespective of patients’ gender due to small numbers; Alt, alternative base; AlphaMissense-based proteome-wide based pathogenicity predictions were performed irrespective of patients’ sex (DOI: 10.1126/science.adg7492); AMP/ACMG, American College of Medical Genetics/Association for Molecular Pathology (see Methods section); Genome position (chromosome X), genome position on chromosome X; gnomAD database (v.4.1.0), frequency per respective sex; hVUS, hot VUS classification (doi: 10.1093/pch/pxab070); Mean GALA conc, mean GALA enzyme concentration in DBSs in male patients (µmol/l/h); M/F, male/female patients; Mean GALA activity (%), mean GALA enzyme activity in % of the norm in males; NA, not applicable; No., number of cases; Normal values, GALA > 15.3 µmol/l/h; lyso Gb3 < 1.8 ng/ml (see Methods section); Ref, reference. Odds ratio calculations were carried out irrespective of the patient's sex base.

### Frequency and classification of the detected GLA variants

*GLA* sequencing identified a total of 39 cases, with variant-positive cases having MFR of 12/27. Of these, the most prevalent variant (*N* = 28) c.937G>T (p.Asp313Tyr) was classified as likely benign (LB) (AMS = 0.1505). It was responsible for the MFR (8/20) in *GLA* variant-positive cases, with mean GALA activity in males at 68% of the norm. The second most common variant (*N* = 4) c.1181T>C (p.Leu394Pro; chrX-101397918-A-G) was classified by us as a “5-point/hot” variant of uncertain significance (VUS) with an AMS = 0.3679 and has an “opposite” 3/1 MFR, with a mean residual GALA activity at 25% in males (Table 3).

All remaining *GLA* variants were classified as LB, each occurring in a single female patient, except for c.1181T>G (p.Leu394Arg), affecting the same codon as the p.Leu394Pro in a male case having GALA activity at 69% of normal values. Since artificial intelligence (AI)-based AlphaMissense (AMS) scores of all of these *GLA* variants were <0.3 and they were absent in the national population database (ref. Supplementary Material for ACGT), except for the c.755G>C (p.Arg252Thr) variant, these could be confidently considered incidental findings (Table 3).

### Enrichment of GLA variant-positive cases compared to the gnomAD database

Observed *GLA* variant frequencies were compared gender-specific to their frequencies in the latest version of the gnomAD database (v.4.1.0), while OR ratios were carried out irrespective of patient gender. Only the four p.Leu394Pro-bearing cases exhibited significant enrichment relative to the current gnomAD v.4.1.0 [OR = 17.78, 95% CI (3.27, 97.1)] (Table 2). ORs for all other *GLA* variants relative to gnomAD were not significant, except for the c.335G>A (p.Arg112His) and c.755G>C (p.Arg252Thr) variants, where non-significant trends were noted in the absence of elevated lyso-Gb3 levels. At the same time, only the latter allele was present in the national ACGT.cz population genomics database (Table 2). Therefore, we referred the p.Leu394Pro screening-positive cases to the collaborating Prague FD center for further *in vitro*, clinical, and laboratory analyses since bioinformatic analyses suggested its association with FD pathogenesis.

## GENOTYPE–PHENOTYPE CORRELATIONS IN THE *GLA* VARIANT-POSITIVE COHORT

### Male-to-female ratios

We also statistically compared the demographic and clinical data of 35 patients bearing the seven *GLA* variants classified as LB (*N* = 35; Table 2), except for the four p.Leu394Pro variants (see above), with a 9/26 MFR, versus 6317 cases from the entire SC (6532 − 35 *GLA* variant-positive cases). The difference in MFR regarding the over-representation of females with LB *GLA* variants was significant (*P* < .0001). Even if all 39 variant *GLA* variant-positive cases were assessed together with the SC (i.e. 6352 − 39 = 6313), with a 12/27 MFR, the ‘skewed’ MFR remained significant (*P* < .000029).

### Age distribution

The mean age of the 35 cases with LB *GLA* variants was significantly higher (71.0 ± 8.4 years) compared to the mean age (64.6 ± 11.2 years) of the SC (6317 cases) (*P* < .0047). This difference remained significant when all *GLA* variant-positive cases were compared with SC (*P* < .00345). Moreover, the mean age of incident RDP cases of both genders in 2017 [12] was 67.1 ± 15.2 years, while in the studied variant group, their mean age when entering KRT was 63.3 ± 8.6 years (range 34–83 years).

### Distribution of primary renal diseases

The distribution of PRD was not significantly different for all 39 *GLA* variant-positive cases compared to the entire SC (data available upon request). All *GLA* variant-positive cases were listed in the RDP as suffering from PRD other than FD. The PRDs most frequently listed in this group are shown in Fig. 1. We also observed higher mortality in the 35 cases with LB *GLA* variants compared to the RDP cohort (40.0% versus 22.7%, respectively) since follow-ups of studied patients were performed prospectively: 3–5 years after sampling. By contrast, deceased RDP patients were reported in a given year, that is, not as follow-up data (Table 3). In the p.Leu394Pro subgroup, two cases were listed as DKD: one due to atherosclerotic renal disease and one as ‘other’ PRD.

### Clinical and laboratory parameters

Clinical data drawn from RDP in the 39 *GLA* variant-positive cases were examined and compared to all CKD cases reported to RDP. Twelve patients (31%) were males and 27 (69%) were females, with a median age of 72.1 years (mean age of 70.9 ± 7.8 years). During the study period, all patients underwent HD-MDT. However, before the commencement of the project, one of them (with variant c.937G>T, p.Asp313Tyr) underwent PD and was switched to HD after 14 months. Finally, we also analyzed the p.Leu394Pro variant subgroup. Besides their “KRT standard” 3/1 MFR and higher mean age, none have undergone KTx thus far, while one patient died during follow-up (Table 3).

### Analysis of the follow-up period

During the follow-up period, the median dialysis treatment vintage for all 39 *GLA* variant-positive cases was 2711 days (almost 7.4 years), with a mean of 2859 days (±939). Twenty patients (51%) survived dialysis treatment, four patients (10%) underwent successful KTx, and 15 patients (39%) died. However, there was a significant difference (*P* < .00792) in the median duration of dialysis treatment between the p.Leu394Pro variant subgroup (median 2039 days) and the 35 cases with the remaining LB variants (2711 days; see also Supplementary Material).

## DISCUSSION

In the current study, we wanted to assess whether, despite the general FD awareness among other specialists (e.g. internists, nephrologists, and cardiologists), facilitated by continuous specialist educational activities of the FD center in Prague, cases of undiagnosed FD in ESKD still exists. We also wanted to assess the logistics of the country-wide availability of DBS-based screening and evaluate the screening utility of the combination of two FD-related biomarkers (GALA and/or lyso-Gb3 levels), i.e. not only GALA as in the first study [10] where six unidentified cases were ascertained. In addition, we carried out sequencing of the entire *GLA* gene in all females and screening-positive males. This way, we also wanted to uniformly assure equitable screening of both males and females for, e.g. the late-onset/atypical forms of FD where GALA concentrations may not be sensitive enough or where *GLA* sequencing generates VUS.

We also aimed to evaluate whether the pathogenic potential of detected *GLA* missense variants could be resolved via their cohort-specific frequency enrichment approaches vis-à-vis sizable population variant cohorts such as the international Genome Aggregation Database (gnomAD; gnomad.broadinstitute.org) and the newly developed Czech population database ACGT.cz. Furthermore, we also applied the novel bioinformatic artificial intelligence (AI)-based algorithms to assess the pathogenicity of detected missense variants in the *GLA* gene at the protein level.

In the second nationwide study, laboratory analyses rendered normal also-Gb3 biomarker levels in all ascertained subjects (males and females), excluding the classical form of FD in the screened ESKD cohort. Hence, typical or close-to-normal values detected in all subjects carrying LB *GLA* variants render their causal association with FD, defined by the degree of lysosomal storage, much less probable.

However, in two male subjects, GALA activities were reduced to 68% and 69%, respectively (Table 3), and in the case of the p.Leu394Pro variant-bearing cases, these were at the “residual level” of 25% of normal values. Although the reduction of GALA activity is still debated as a potential, yet unconfirmed, risk factor aggravating other pathologic conditions and mechanisms leading to ESKD [18], differences between the two biomarkers across the entire SC were insignificant, and thus suggest another potentially FD-related pathogenetic mechanism. All the 39 detected *GLA* missense variants (in males and females) are classified as LB and, except in the hot VUS—p.Leu394Pro in four patients (three males and one female) with ESKD with males having the aforementioned residual GALA activity (Table 3).

Moreover, our observation is in sharp contrast to the meta-analysis by Doheny *et al.* [7] on the reassessment of the pathogenicity of *GLA* variants previously detected in other studies using the International Fabry Disease Genotype/Phenotype Database (FabryGP; fabrygenphen.com). This meta-analysis used the FabryGP “binary” variant classification (assigning merely “pathogenic” and “benign” variants), thereby precluding proper comparison to the AMP/ACMG classification in the current screening scheme (ref. Supplementary Material).

An intriguing finding was the significant enrichment of the hot VUS p.Leu394Pro relative to the gnomAD, together with its “standard” MFR in terms of ESKD cases in RDP (Tables 2 and 3). This variant was also not present in the ACGT.cz database or listed in the FabryGP database. However, (i) owing to the high degree of renal involvement in ESKD patients bearing this variant, (ii) earlier entry into KRT, (iii) significantly longer dialysis treatment compared to cases bearing other *GLA* variants (Table 3), and (iv) the finding of other individuals in pre-HD in their genetic relatives, indirectly indicated its implication in alternative FD pathogenesis.

This observation triggered comprehensive analyses of the hot VUS p.Leu394Pro by our collaborators at the Prague FD center, who carried out further *in vitro*, clinical, laboratory, and family analyses [19–21]. These studies identified defective proteostasis of mutated GALA, resulting in chronic endoplasmic reticulum stress and unfolded protein response of GALA expressing cells, as contributors to FD pathogenesis. This allele stems from a population-specific founder effect as demonstrated by corroborating genetic and family studies [19]. Interestingly, we found that at the same amino acid position, another LB variant c.1181T>G (p.Leu394Arg) with AMS = 0.1617 was found in an 80-year-old male patient from the SC, with altered GALA enzyme activity (69%) but normal lyso-Gb_3_ level (mean level of 1.1 ng/ml) (Table 3).

### Genotype–phenotype correlations in GLA variant-positive cases

There was a significantly higher percentage of females in the cohort of 35 cases with LB *GLA* variants than in the overall SC. This value was significant even when we compared the entire SC minus the *GLA* variant-positive instances (Table 3). The higher prevalence of LB variants in the *GLA* could be explained by the fact that these could “accumulate” during evolution in females who have two copies of the gene on the X chromosome compared to “hemizygous” males (see the FabryGP database). The *GLA* variants observed in females are most likely not associated with FD-related pathogenesis of ESKD. Furthermore, their prevalence was more consistent with that in the general population, as proved by comparing their frequencies to the gnomAD and ACGT databases. In other words, a female is twice as likely to have a *GLA* variant as a male (Table 2), except for p.Leu394Pro, which is expected to be associated with alternative pathogenesis of FD and is thus more likely to manifest in hemizygous males. The presence of a single female in this small cohort could be due to non-random X chromosome inactivation patterns [22]. The comparable MFR of the p.Leu394Pro variant sub-cohort vis-à-vis the RDP cohort provides an additional indirect line of evidence for its implication in alternative pathogenesis in FD.

Interestingly, other *GLA* variant-positive cases enter the KRT at a significantly older age because their ESKD is most likely not mediated by FD-related pathogenesis, as confirmed by the distribution of PRD diagnoses that do not differ from those in the RDP. Thus, the *GLA* 35 LB variants represent an SC-related incidental finding that would otherwise have gone unnoticed.

Hence, compared to the 2002 Czech nationwide screening program, we did not detect a single misdiagnosed case of FD in ESKD, which could be due to the “classical” lysosome dysfunction-based pathogenesis that could be laboratory-substantiated by altered GALA activity and/or lyso-Gb_3_ biomarkers (Tables 2 and 3). This achievement is likely due to the generally improved clinical and laboratory testing for FD within the last decade and a half in our country, including increased awareness of complex FD symptoms among collaborating physicians who indicate suspicious cases for further examination at specialized national FD centers. Confirmed cases of FD are then treated with enzyme replacement therapy (ERT), which National Health Insurance fully covers. Thus, these patients did not undergo KRT due to the underdiagnosis of FD.

Our FD screening results highlight the importance of a comprehensive, multidisciplinary clinical approach, including a thorough evaluation of medical history and early suspicion of FD in patients with unexplained progressive CKD. Appropriate diagnostic procedures, including RB, should be followed to optimize early diagnosis and prevent progression to ESKD in undiagnosed cases. A structured nationwide diagnostic framework within the healthcare system is critical when full access to ERT is available. However, given the costs of laboratory testing, logistical challenges, and low prevalence of pathogenic *GLA* variants, we shall reassess the underdiagnosis of FD in ESKD in ∼10 years’ time to account for changes in the population demographics and potential consolidation of dialysis units within the ongoing healthcare system reform.

### Strengths and limitations

We screened a large representative cohort with more sensitive and specific laboratory examinations than most previously published screening cohorts in European populations, including our previous nationwide scheme. Another strength is the application of state-of-the-art bioinformatics approaches to *GLA* variant classification and “variant-annotation” with complex demographic, clinical, and laboratory data drawn from the nationwide RDP.

We acknowledge that many patients were detected with a *GLA* variant but declined further clinical and laboratory investigations. Therefore, clinical data regarding the potential and/or non-renal multisystemic manifestations of FD pathogenesis are incomplete, except for four cases and their families with the p.Leu394Pro variant who were subjected to thorough follow-up at the Prague FD center. Since most KRT patients had not undergone RB, histologic evidence of lysosomal storage is unavailable.

## CONCLUSIONS

The second nationwide FD screening study in cases of ESKD represents one of the most significant population-specific projects carried out in a representative and homogeneous European population [23]. We identified only one potentially pathogenic *GLA* variant in four cases, where the pathogenesis of ESKD was due to alternative pathogenesis based on chronic endoplasmic reticulum stress and unfolded protein response. The remaining *GLA* variants represented SC-related incidental findings. Finally, we provide evidence that raising awareness of the complex symptomatology of FD in other collaborating medical specialties and a long-term nationwide multidisciplinary collaboration represents an efficient strategy for diminishing the underdiagnosis of FD in ESKD.

## Supplementary Material

sfaf167_Supplemental_Files

## Data Availability

The data supporting this study's findings are not publicly available because of information that could compromise the privacy of research participants. Still, they are available from the corresponding author, Prof. Ivan Rychlík, MD, PhD, upon request. Additional general data about the Czech dialysis patients were derived from the Registry of Dialysis Patients of the Czech Republic, which is available in the public domain at [https://www.nefro.cz/].
