# Supplementary material for: Czech nationwide screening for Fabry disease in patients on maintenance dialysis: a call for evaluation of population-enriched GLA gene variants of uncertain significance
Source: Clin Kidney J. 2025 May 28;18(6):sfaf167. doi: 10.1093/ckj/sfaf167 (PMC12202863; doi:10.1093/ckj/sfaf167)
Supplement: sfaf167_Supplemental_Files [file sfaf167_supplemental_files.zip › 1573 Online Supplementary Material.docx]

**Online Supplementary Material**

*FD screening*

Peripheral blood was drawn and applied to proprietary filtration paper (CentoCard; CentoGene AG, Germany; www.centogene.com). DBS samples were dried for several hours, and stored in a plastic sleeve (both at room temperature) for no more than a week. In males, the enzymatic activity of GALA and concentration of the lyso-Gb_3_ biomarker were determined using fluorimetry and liquid chromatography-mass spectrometry, respectively [1]. If the activity of GALA has decreased below 15.3 µmol/L/hour and the concentration of lyso-Gb_3_ increased over 1.8 ng/ml, then the entire G*LA* (reference: NM_000169.2) was sequenced using the massively parallel locus-specific approach on the NextSeq550 platform (Illumina.com; USA).

Given the high rate of expected false-negative results of biochemical assays in females, initially, *GLA* sequencing was initially performed. Only once a variant, irrespective of its AMP/ACMG pathogenic class, was detected was an examination of lyso-Gb_3_ performed. The positive outcomes of FD screening were referred to the specialized FD Center for further clinical and laboratory investigations and management, including family screening/diagnosis. Statistical and bioinformatic analyses are described in the supplementary material.

*Statistical and bioinformatic analyses*

The demographic, clinical, and laboratory parameters were corrected for multiple comparisons. We used the Mann–Whitney U test for continuous variables and the χ2 test or Fisher’s exact test for noncontinuous variables. For comparison of *GLA* variant frequencies, we used Fisher’s exact test. P was set than 0.05, using IBM SPSS Statistics 25 (IBM; USA) and STATA IC 16.1 (StataCorp, USA) software.

Furthermore, we used the odds ratio (OD) test [2] to assess the enrichment of the identified *GLA* variants in the SC versus the Genome Aggregation Database (gnomAD; v4.0.0. gnomad.broadinstitute.org) as the control population for OD computation of variant frequencies. A representative Czech genome variant database (database/acgt. cz; ACGT) is still small and was used only for general information on detected *GLA* variant frequencies. A variant with an OR >5 and a confidence interval (CI) around the OR (not including 1) was considered significantly enriched. The pathogenicity of missense *GLA* variants was assessed using the proteome-based AlphaMissense variant effect predictor (henceforward we report the AMS score) [3]. The AMP/ACMG classification was used, and the respective terminology was applied. For variants of uncertain significance (VUS), we added a natural scaled point system using the Bayesian framework [4] on VarSome.com.

We consider the “remaining” 6313 *GLA* variant-negative cases in the SC as being representative (i.e., accounting for over 93% of the RDP dataset from within the 2016-2021 period) because the RDP database incorporates compulsory case anonymization in terms of the follow-up outcomes.

*Predictions of GLA variant pathogenicity using the AMP/ACMG classification, including their presence in FabryGP*

A meta-analysis by Doheny et al. [5] showed that a large proportion of the identified *GLA* variants were not responsible for ESKD because they were not associated with significant enzymatic defects in FD. These variants are currently considered variants of uncertain significance (VUS), LB, or benign according to the American College of Medical Genetics/Association for Molecular Pathology (AMP/ACMG) variant classification [6]. After revision of the current pathogenicity criteria for FD, the prevalence of pathogenic GLA variants has decreased to 0.21% and 0.15% in males and females with ESKD, respectively.

The authors also utilized for patient stratification Class I “early” and milder course of the FD versus Class II “later” and less severe onset of FD, with the age cut-off of 30 years [5]. The overall frequency of “pathogenic” variants, regardless of the patient´s sex, reached 0.18%, which is approximately ten times higher than the frequency we observed. However, the total observed *GLA* variants in SC, irrespective of their pathogenic classification, yielded an overall frequency of 0.61%, which is comparable to the 0.51% reported by the aforementioned meta-analysis. This observation corroborates the obvious bias due to the incomparable *GLA* variant classifications. Although we cannot render a more precise assessment of retrospective *GLA* variant data, we could evaluate the observed 7 types of *GLA* variants (Table 2) vis-a-vis their classification in the FabryGP [7] and provide a current literature review.

Currently, FabryGP contains more than 900 *GLA* variants [7]. Most of these are rare or even private in families affected by FD. FabryGP also clearly states that the presence of a *GLA* variant does not “automatically” implies the diagnosis of FD. Moreover, FabryGP distinguishes whether a *GLA* variant is “pathogenic” (based on a database-specific “flowchart”) and whether it is associated with a classical or non-classical form of FD. Such a distinction is prognostically relevant and can be applied for therapeutic guidance and/or genetic counselling. In this regard, there are: a) male patients with a classical “sever” FD developing before 30 years of age with GALA activity substantially reduced or absent (less than 5% of the mean of the reference range); b) male patients with non-classical and “less severe” FD (late-onset FD or atypical FD) where the characteristic childhood/adolescent symptoms not fully pronounced, including residual GALA activity; c) in females classical FD is more akin to that in males described in point b) and c) females with non-classical FD usually have a late onset and mild course of the disease and could even be asymptomatic. In the latter case, the GALA level was reduced or normal.

The c.335G>A (p.Arg112His) variant, which we classified as LB (AMS=0.2727) (Table 2), remains controversial in terms of its pathogenicity, with several laboratory reports reporting it as likely pathogenic, while others are more inclined to classify it as benign or LB [8]. Our patient was an 87-year-old woman who declined further investigations, as did her two apparently healthy sons. Her lyso-Gb_3_ level was not elevated to 1.5 ng/ml. This variant (dbSNP: rs372966991) is present in 0.0024% (2/81850) of the non-Finnish European chromosomes in the gnomAD database and was described in 15 individuals with FD and segregated with the disease in two families [9-18]. The p.Arg112His variant is located in a region of *GLA* that is essential for GALA protein folding and thus likely affects its stability, thereby supporting its pathogenicity [19,20]. Two additional missense pathogenic variants, causing a different amino acid change at the same position (p.Arg112Cys, p.Arg112Leu), have been reported in association with FD in the literature and the ClinVar database (Variation IDs: 92550; 92551), suggesting that a change in this GALA protein position is likely deleterious [21-23]. The FabryGP database contains information on one male with classical FD, nine males with non-classical FD, and ten females with non-classical FD, generally corroborating the above-listed clinical and laboratory observations/classifications.

Two of our female patients (80 and 73 years old) carried the c.352C>T (p.Arg118Cys) variant, which was classified as LB (Table 2). However, in both cases, the lyso-Gb_3_ biomarker level was not elevated, and family members were lost to follow-up or declined further clinical/laboratory examinations. This variant was also described elsewhere in the literature [24-28], whereby Spada *et al.* [24] suggested that it does not interfere with the active site of the GALA enzyme but may rather be associated with its altered stability. The authors suggested that this may be associated with the later-onset manifestation of FD. Accordingly, it has been reported in an adult female patient with apical hypertrophic cardiomyopathy (HCM) and reduced GALA activity. However, another group of authors [29] reported data from an in-depth review of individuals of Portuguese and Spanish ancestry bearing this variant and suggested an unclear association with FD; however, the authors did not rule out that it could “modulate” the risk of developing FD. This variant was identified in male controls, but its clinical details are sketchy [30]. The FabryGP database lists one instance, clearly indicating its benign character, following the literature discussion above.

The variant c.376A>G (p.Ser126Gly), which we classified as LB (Table 2), was found in a 70-year-old female who succumbed to complications of ESKD before being investigated by us, with her nine genetic relatives testing negative. Her lyso-Gb_3_ levels were low (1.3 ng/ml). The variant has been repeatedly described as benign, LB, or as a VUS with a mild reduction in residual enzyme activity by approximately 50% [31-33]. It was previously reported in 0.074% of European-derived general populations [34], but was absent in the ACGT database. The reported associations with potential FD manifestations are heterogeneous and often unclear nature [31-33]. The predictive model developed to determine the pathogenicity of variants in *GLA* suggests that the p.Ser126Gly variant may not be damaging after all [35], as independently substantiated by a low AMS of 0.0737. However, the FabryGP database does not include this variant.

The variant c.427G>A (p.Ala143Thr), classified as LB by us (Table 2), was found in a 44-year-old female. This variant is frequently identified in high-risk population FD screening programs, including neonatal screening schemes [24]. The variant was present in a large population cohort at a frequency of 0.05% (rs104894845; 104/205433 chromosomes; 0 homozygotes; and 29 hemizygotes in the gnomAD database). However, some authors classify it as pathogenic, albeit mostly associated with late-onset FD manifestations [36]. Recent publications [15, 37] suggest that most cases with this variant have only a modest decrease in GALA activity, together with a normal or minimally elevated lyso-Gb3 level. Pedigree analysis of probands´ families usually does not prove the association of this variant with FD. Moreover, kidney biopsies of the p.Ala143Thr variant showed no signs of significant Gb_3_ lysosomal accumulation [38]. However, cases with elevated lyso-Gb_3_ levels and typical late-onset FD findings have also been identified [36]. Therefore, most literature resources classify p.Ala143Thr as LB or VUS, as corroborated by our bioinformatics analyses with AMS = 0.1608. The FabryGP database lists 4 male and 11 female instances, all classified as benign.

The variant c.755G>C (p.Arg252Thr), classified as LB by us (Table 2), was identified in a 79-year-old female. This variant is also present in the ACGT population databases, and previous functional analyses classified it as benign [23], as corroborated by our low AMS of 0.0581. However, the FabryGP database does not include this variant.

The most commonly observed *GLA* variant was c.937G>T (p.Asp313Tyr). We found 28 (0.44%) carriers, which is comparable to the 0.5% reported among European-derived populations [39] and 0.42% among patients with ESKD [40]. However, data on its pathogenicity and clinical relevance remain controversial. Carriers of the p.Asp313Tyr variant did not manifest the classical multiorgan early onset FD phenotype. A recent meta-analysis by Effraimidis et al. [40] confirmed the high residual enzyme activity and absence of a significant increase in either lyso-Gb_3_, including minimal or absent intracellular Gb_3_ accumulation in skin and kidney biopsies. Its presence in the ACGT database and AMS=0.1505 further support this notion (Table 2). The FabryGP database lists this variant as benign in 4 male and 2 female cases.

*Additional information on the Analysis of the follow up period*

Only two patients underwent RB. One female bearing *the GLA* variant c.937G>T(p.Asp313Tyr) underwent RB in 2008. Histological examination revealed hypertensive nephrosclerosis with secondary focal segmental glomerulosclerosis (FSGS). In the other male patient, bearing the p.Leu394Pro variant, RB was performed in 2011 and confirmed nodular diabetic glomerulosclerosis. In 2017, a second reading was performed, which confirmed the former findings and found no signs of FD, including insignificant lyso-Gb_3_ values.

Concerning other clinical comorbidities, arterial hypertension was reported in 22 cases (57%) and diabetes mellitus type 2 in 13 patients (33%) in the RDP. Of interest are the results concerning the start of KRT at a younger age (defined as entering dialysis at <60 years of age), which was the case in 5 patients (13%). Four of them had the c.937G>T (p.Asp313Tyr) variant, while one had the c.427G>A p.(Ala143Thr) variant. All of them were females with the youngest KRT entry at the age of 34 years.
