# Supplementary material for: Czech nationwide screening for Fabry disease in patients on maintenance dialysis: a call for evaluation of population-enriched GLA gene variants of uncertain significance
Source: Clin Kidney J. 2025 May 28;18(6):sfaf167. doi: 10.1093/ckj/sfaf167 (PMC12202863; doi:10.1093/ckj/sfaf167)
Supplement: sfaf167_Supplemental_Files [file sfaf167_supplemental_files.zip › 15Supplementary References.docx]

**Supplementary References**

1/ Aerts JM, Groener JE, Kuiper S, Donker-Koopman WE, Strijland A, Ottenhoff R, van Roomen C, Mirzaian M, Wijburg FA, Linthorst GE, Vedder AC. Elevated globotriaosylsphingosine is a hallmark of Fabry disease. Proceedings of the National Academy of Sciences. 2008;105(8):2812-7.

2/ Walsh R, Mazzarotto F, Whiffin N, et al. Quantitative approaches to variant classification increase the yield and precision of genetic testing in Mendelian diseases: the case of hypertrophic cardiomyopathy. Genome Med 11, 5 (2019). https://doi.org/10.1186/s13073-019-0616-z

3/ Cheng J, Novati G, Panet J, et al. Accurate proteome-wide missense variant effect prediction with AlphaMissense. Science 2023; 381: 1303. DOI: 10.1126/science.adg7492

4/ Tavtigian SV, Harrison SM, Boucher KM, Biesecker LG. Fitting a naturally scaled point system to the ACMG/AMP variant classification guidelines. Hum Mutat. 2020; 41: 1734-1737. doi: 10.1002/humu.24088. PMID: 32720330; PMCID: PMC8011844

5/ Doheny D, Srinivasan R, Pagant S, Chen B, Yasuda M, Desnick RJ. Fabry disease: prevalence of affected males and heterozygotes with pathogenic *GLA* mutations identified by screening renal, cardiac, and stroke clinics, 1995–2017. Journal of medical genetics. 2018;55(4):261-8.

6/ Masson, E., Zou, WB., Génin, E. et al. Expanding ACMG variant classification guidelines into a general framework. Hum Genomics 16, 31 (2022). https://doi.org/10.1186/s40246-022-00407-x
https://humgenomics.biomedcentral.com/articles/10.1186/s40246-022-00407-x

7/ Fabry-Gen-Phen: The Fabry Working Group Genotype-Phenotype Database. http://fabrygenphen.com/ accessed March 25^th^ 2023

8/ B E Smid BE, Hollak CEM, Poorthuis BJHM, et al. Diagnostic dilemmas in Fabry disease: a case series study on GLA mutations of unknown clinical significance. Clin Genet 2015; 88:161-6.  doi: 10.1111/cge.12449. Epub 2014 September 5.

9/ Eng CM, Desnick RJ. Molecular basis of Fabry disease: mutations and polymorphisms in the human α‐galactosidase A gene. Human mutation. 1994;3(2):103-11.

10/ Schäfer E, Baron K, Widmer U, Deegan P, PH Neumann H, Sunder‐Plassmann G, Johansson JO, Whybra C, Ries M, M. Pastores G, Mehta A. Thirty‐four novel mutations of the *GLA* gene in 121 patients with Fabry disease. Human Mutation. 2005;25(4):412-.

11/ Shimotori M, Maruyama H, Nakamura G, Suyama T, Sakamoto F, Itoh M, Miyabayashi S, Ohnishi T, Sakai N, Wataya‐Kaneda M, Kubota M. Novel mutations of the *GLA* gene in Japanese patients with Fabry disease and their functional characterization by active site-specific chaperone. Human mutation. 2008;29(2):331-.16/ Sirrs S, Clarke JT, Bichet DG, Casey R, Lemoine K, Flowerdew G, Sinasac DS, West ML. Baseline characteristics of patients enrolled in the Canadian Fabry Disease Initiative. Molecular genetics and metabolism. 2010;99(4):367-73.

12/ Gaggl M, Kain R, Jaksch P, Haider D, Mundigler G, Voigtländer T, Sunder-Plassmann R, Rommer P, Klepetko W, Sunder-Plassmann G. A single lung transplant in a patient with Fabry disease: causality or far-fetched? A case report. Case Reports in Transplantation. 2013;2013.

13/ Nishida M, Kosaka K, Hasegawa K, Nishikawa K, Itoi T, Tsukimura T, Togawa T, Sakuraba H, Hamaoka K. A case of Fabry nephropathy with histological features of oligo nephropathy. European journal of pediatrics. 2014;173:1111-4.

14/ Sechi A, Nucifora G, Piccoli G, Dardis A, Bembi B. Myocardial fibrosis as the first sign of cardiac involvement in a male patient with Fabry disease: report of a clinical case and discussion on the utility of the magnetic resonance in Fabry pathology. BMC Cardiovascular Disorders. 2014;14(1):1-5.

15/ Smid BE, Hollak CE, Poorthuis BJ, Van den Bergh Weerman MA, Florquin S, Kok WE, Lekanne Deprez RH, Timmermans J, Linthorst GE. Diagnostic dilemmas in Fabry disease: a case series study on *GLA* mutations of unknown clinical significance. Clinical genetics. 2015; 88(2):161-6.

16/ Arends M, Wanner C, Hughes D, Mehta A, Oder D, Watkinson OT, Elliott PM, Linthorst GE, Wijburg FA, Biegstraaten M, Hollak CE. Characterization of classical and non-classical Fabry disease: a multicenter study. Journal of the American Society of Nephrology. 2017;28(5):1631-41.

17/ Van der Tol L, Sminia ML, Hollak CE, Biegstraaten M. Cornea verticillata supports a diagnosis of Fabry disease in non-classical phenotypes: results from the Dutch cohort and a systematic review. British Journal of Ophthalmology. 2016;100(1):3-8.

18/ Nampoothiri S, Yesodharan D, Bhattacharjee A, Ahamed H, Puri RD, Gupta N, Kabra M, Ranganath P, Bhat M, Phadke S, Radha Rama Devi A. Fabry disease in India: A multicenter study of the clinical and mutation spectrum in 54 patients. JIMD reports. 2020;56(1):82-94.

19/ Kuchar L, Berna L, Poupetova J, Ledvinova J, Ruzicka P, Dostalova G, Reichmannova S, Asfaw B, Linhart A, Sikora J. LysoGb3 quantification facilitates phenotypic categorization of Fabry disease patients: Insights gained by a novel MS/MS method. Clin Chim Acta 2024 July 15:561:119824. doi: 10.1016/j.cca.2024.119824

20/ Ishii S, Chang HH, Kawasaki K, Yasuda K, Wu HL, Garman SC, Fan JQ. Mutant alpha-galactosidase A enzymes identified in Fabry disease patients with residual enzyme activity: biochemical characterization and restoration of normal intracellular processing by 1-deoxygalactonojirimycin. Biochem J. 2007 September 1;406(2):285-95. doi: 10.1042/BJ20070479. PMID: 17555407; PMCID: PMC1948963.

21/ Ishii S, Sakuraba H, Suzuki Y. Point mutations in the upstream region of the alpha-galactosidase A gene exon 6 in an atypical variant of Fabry disease. Hum Genet. 1992 April;89(1):29-32. doi: 10.1007/BF00207037. PMID: 1315715.

22/ Yasuda M, Shabbeer J, Benson SD, et al. Fabry disease: characterization of alpha-galactosidase A double mutations and the D313Y plasma enzyme pseudodeficiency allele. Human Mutation. 2003 December;22(6):486-492. DOI: 10.1002/humu.10275. PMID: 14635108.

23/ Lukas J, Giese AK, Markoff A, Grittner U, Kolodny E, Mascher H, Lackner KJ, Meyer W, Wree P, Saviouk V, Rolfs A. Functional characterization of alpha-galactosidase mutations as a basis for a new classification system in Fabry disease. PLoS Genet. 2013;9(8):e1003632. doi: 10.1371/journal.pgen.1003632. Epub 2013 August 1. PMID: 23935525; PMCID: PMC3731228.

24/ Spada M, Pagliardini S, Yasuda M, Tukel T, Thiagarajan G, Sakuraba H, et al. High incidence of later-onset Fabry disease revealed by newborn screening*. The American Journal of Human Genetics. 2006;79(1):31–40.

25/ Morais P, Santos ALÍ, Baudrier T, Mota AV, Oliveira JP, Azevedo F. Angiokeratomas of Fabry successfully treated with intense pulsed light. Journal of Cosmetic and Laser Therapy. 2008;10(4):218–22.

26/ Baptista MV, Ferreira S, Pinho-e-Melo T, Carvalho M, Cruz VT, Carmona C, et al. Mutations of the *GLA* gene in young patients with stroke. Stroke. 2010;41(3):431–6.

27/ Gaspar P, Herrera J, Rodrigues D, Cerezo S, Delgado R, Andrade CF, Forascepi R, Macias J, del Pino MD, Prados MD, de Alegria PR, Torres G, Vidau P, Sá-Miranda MC. Frequency of Fabry disease in male and female hemodialysis patients in Spain. BMC Med Genet. 2010 February 1;11:19. doi: 10.1186/1471-2350-11-19. PMID: 20122163; PMCID: PMC2837018.

28/ Pasqualim G, Simon L, Sperb-Ludwig F, Burin MG, Michelin-Tirelli K, Giugliani R, et al. Fabry disease: A new approach for the screening of females in high-risk groups. Clinical Biochemistry. 2014;47(7-8):657–62.

29/ Caetano F, Botelho A, Mota P, Silva J, Leitão Marques A. Fabry disease presenting as apical left ventricular hypertrophy in a patient carrying the missense mutation R118C. Revista Portuguesa de Cardiologia. 2014;33(3).

30/ Ferreira S, Ortiz A, Germain DP, Viana-Baptista M, Caldeira-Gomes A, Camprecios M, et al. The alpha-galactosidase a P.ARG118CYS variant does not cause a Fabry disease phenotype: Data from individual patients and Family Studies. Molecular Genetics and Metabolism. 2015;114(2):248–58.

31/ Branton MH, Schiffmann R, Sabnis SG, Murray GJ, Quirk JM, Altarescu G, Goldfarb L, Brady RO, Balow JE, Austin Iii HA, Kopp JB. Natural history of Fabry renal disease: influence of α-galactosidase A activity and genetic mutations on clinical course. Medicine. 2002 March 1;81(2):122-38.

32/ Colon C, Ortolano S, Melcon-Crespo C, Alvarez JV, Lopez-Suarez OE, Couce ML, Fernández-Lorenzo JR. Newborn screening for Fabry disease in the north-west of Spain. European journal of pediatrics. 2017 August;176:1075-81.

33/ Reisin RC, Mazziotti J, Cejas LL, Zinnerman A, Bonardo P, Pardal MF, et al. Prevalence of Fabry disease in young patients with stroke in Argentina. Journal of Stroke and Cerebrovascular Diseases. 2018;27(3):575–82.

34/ Samuelsson K, Kostulas K, Vrethem M, Rolfs A, Press R. Idiopathic small fibre neuropathy: Phenotype, Etiologies, and the search for Fabry disease. Journal of Clinical Neurology. 2014;10(2):108.

35/ Riera C, Lois S, Domínguez C, Fernandez-Cadenas I, Montaner J, Rodríguez-Sureda V, et al. Molecular damage in Fabry Disease: Characterization and prediction of alpha-galactosidase pathological mutations. Proteins: Structure, Function, and Bioinformatics. 2014;83(1):91–104.

36/ Eng CM, Ashley GA, Burgert TS, Enriquez AL, D’Souza M, Desnick RJ, et al. Fabry disease: Thirty-five mutations in the α-galactosidase gene in patients with classic and variant phenotypes. Molecular Medicine. 1997;3(3):174–82.

37/ Lenders M, Weidemann F, Kurschat C, Canaan-Kühl S, Duning T, Stypmann J, Schmitz B, Reiermann S, Krämer J, Blaschke D, Wanner C. Alpha-Galactosidase A p. A143T, a non-Fabry disease-causing variant. Orphanet Journal of Rare Diseases. 2016; 11:1-9.

38/ Terryn W, Vanholder R, Hemelsoet D, Leroy BP, Van Biesen W, De Schoenmakere G, et al. Questioning the pathogenic role of the *GLA* p.ala143thr “mutation” in Fabry disease: Implications for screening studies and ERT. JIMD Reports. 2012;:101–8.

39/ Phan L, Jin Y, Zhang H, Qiang W, Shekhtman E, Shao D, et al. ALFA: Allele Frequency Aggregator. National Center for Biotechnology Information, US National Library of Medicine 2020.

40/ Effraimidis G, Rasmussen ÅK, Bundgaard H, Sørensen SS, Feldt-Rasmussen U. Is the alpha-galactosidase A variant p.Asp313Tyr (p.D313Y) pathogenic for Fabry disease? A systematic review. *J Inherit Metab Dis*. 2020;43(5):922-933. doi:10.1002/jimd.12240
